# Low‐Frequency, Ultra‐Thin and Ultra‐Wideband Meta‐Absorber via Resonance Enhancement Harnessing Magnetic Material

**DOI:** 10.1002/nap2.70140

**Published:** 2026-05-19

**Authors:** Dan Liu, Xingshuo Cui, Yuxu Chen, Xiongyu Liang, Xiaochun Liu, Zhenxu Wang, Fei Gao, Tong Cai

**Affiliations:** ^1^ State Key Laboratory of Modern Optical Instrumentation the Electromagnetics Academy Zhejiang University Hangzhou China; ^2^ Air Defense and Antimissile School Air Force Engineering University Xi'an China; ^3^ The Research Institute for Special Structures of Aeronautical Composite ACICA The Aeronautical Science Key Lab for High Performance Electromagnetic Windows Ji'nan China

## Abstract

Achieving efficient, ultra‐thin, and broadband absorption in the low‐frequency regime (e.g., P to S bands, 0.23–4 GHz) remains a significant challenge due to the inherent limitations of conventional materials and designs. To address this, we propose a composite metamaterial absorber that synergistically integrates a three‐layer metasurface absorber (TLMA) with a top‐loaded magnetic material (MM) layer. The standalone TLMA achieves perfect absorption (> 90%) but within a narrow 300 MHz band around 0.77 GHz. The introduction of the MM layer drastically enhances and broadens the resonant response through magnetic loss and optimized coupling, yielding an ultra‐wide absorption bandwidth from 0.5 to 3.8 GHz, which effectively covers the P, L, and S bands. The final optimized structure exhibits a remarkable overall thickness of only 41.0 mm (0.068*λ* at the lowest frequency) and a fractional bandwidth of 153.5%. The working mechanism, elucidated through surface current and equivalent impedance analysis, is attributed to magnetic‐material‐assisted resonance optimization. This work provides a practical and effective strategy for developing high‐performance, ultra‐thin absorbers for advanced low‐frequency applications.

## Graphical abstract

This work proposes a novel strategy to integrate ultra‐wideband absorption and ultra‐thin thickness for low‐frequency P–S bands. A composite metamaterial absorber is verified experimentally by synergistically combining a customized three‐layer resonant structure with a top‐loaded magnetic material layer. Assisted by magnetic material optimization of local resonances, the realized absorber achieves over 90% absorption across 0.5–3.8 GHz (fractional bandwidth 153.5%) with an ultra‐thin thickness of merely 0.068λ.

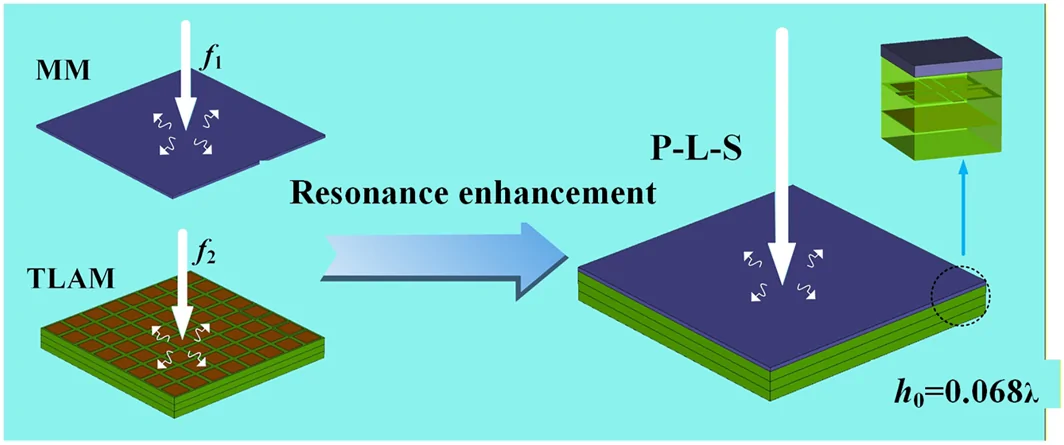

## 1 Introduction

Traditional microwave absorbers, primarily based on transition metals and their oxides [1, 2, 3, 4], conductive polymers [5], and carbon‐based materials [6, 7, 8], have been extensively studied. Although relatively mature, these materials often face a fundamental trade‐off: it is challenging for a single‐layer absorber to simultaneously achieve both ultra‐thin thickness and ultra‐broadband performance. To overcome this limitation, advanced design strategies have been explored, such as incorporating lumped elements [9], employing three‐dimensional architectures [10, 11] and utilizing multi‐layer stacking [12, 13]. These methods can enhance bandwidth but typically at the cost of increased structural complexity and thickness, which becomes particularly prohibitive for applications in the low‐frequency regime.

Meanwhile, the development of nanostructured absorbers and metasurfaces has shown remarkable progress [14, 15, 16, 17, 18, 19, 20, 21]. Nanostructures such as carbon nanotubes and carbon nanocages offer high specific surface areas and rich interfaces, leading to significantly improved absorption efficiency [22, 23, 24, 25, 26, 27, 28]. However, their exceptional performance is predominantly confined to higher frequencies, with limited effectiveness in the low‐frequency spectrum. This is a critical gap, given the growing strategic importance of low‐frequency (e.g., P to S band, 0.23–4 GHz) stealth technology, driven by the development of modern long‐wave radar systems [29, 30, 31, 32, 33, 34]. Recent efforts to address low‐frequency absorption have focused on novel material composites, such as MOF‐derived porous carbons [35, 36, 37, 38] and Fe_3_O_4_‐dielectric hybrids [39, 40, 41, 42]. These materials leverage enhanced impedance matching and synergistic dielectric/magnetic losses, showing great promise for low‐frequency microwave attenuation.

Despite these advances, the integration of ultra‐wideband performance, ultra‐thin profile, and effective low‐frequency operation into a single, practical design remains an outstanding challenge. In this work, we propose and experimentally validate a novel composite metamaterial absorber that successfully bridges this gap. As shown in Figure 1, our design synergistically combines a tailored three‐layer metamaterial absorber (TLMA) with a top‐loaded magnetic material (MM) layer. The TLMA provides a multi‐resonance foundation, whereas the MM layer, characterized by a high imaginary part of permeability (*μ*″), introduces strong magnetic loss and critically enhances the local resonance coupling. This magnetic‐material‐assisted optimization enables the final structure, with a minimal total thickness of 41 mm (0.068λ at the lowest frequency), to achieve an ultra‐wide absorption bandwidth from 0.5 to 3.8 GHz (relative bandwidth of 153.5%) with an efficiency exceeding 90%, effectively covering the P, L, and S bands. This study not only presents a high‐performance absorber but also provides a new design paradigm for overcoming the low‐frequency thickness‐bandwidth constraint in stealth technologies.

**FIGURE 1:**
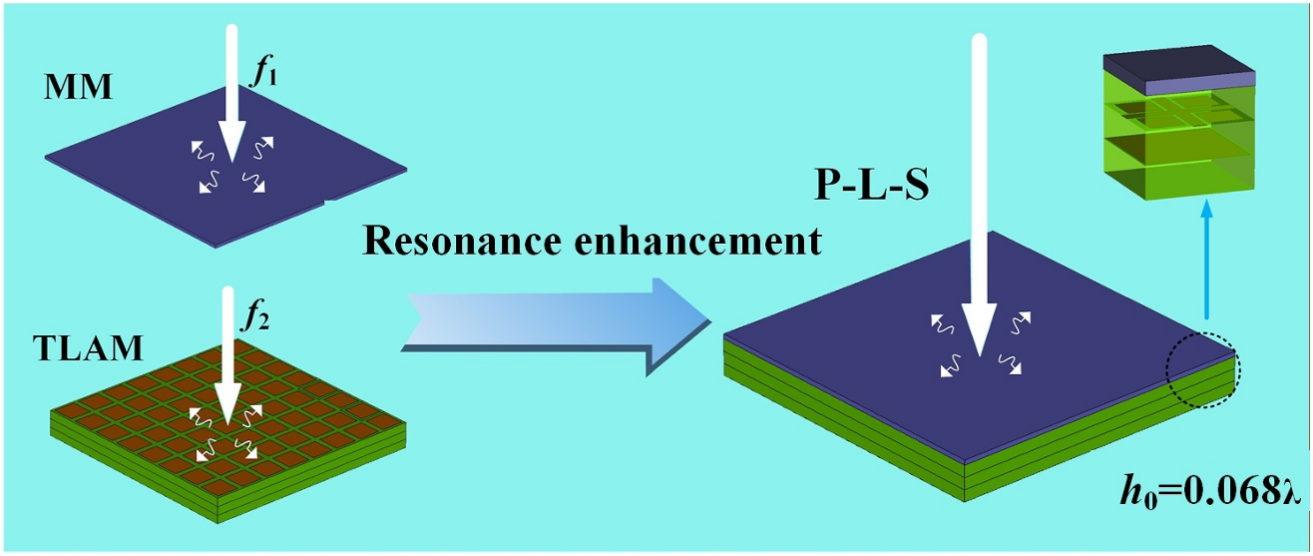
The schematics and working principles of the proposed composite meta‐absorber. The meta‐absorber consists of the upper MM layer and the lower TLMA layer. The MM can enhance the local resonance coupling of the TLMA structure, resulting in a high absorption efficiency covering 0.5–3.8 GHz and an ultra‐thin thickness of 0.068λ.

## 2 Results

Leveraging multiple resonance characteristics, multi‐layered structures are an effective strategy for broadening the absorption bandwidth of metasurfaces [9, 10, 11, 12, 13, 14]. Following this principle, we first designed a TLMA as the foundational component for low‐frequency operation. As illustrated in Figure 2a, the proposed TLMA unit cell comprises four layers. The bottom is a metallic ground plane that completely reflects incident waves, ensuring zero transmission. Above it, there are three identical FR4 dielectric spacers (each with a thickness *h*), each supporting a patterned indium tin oxide (ITO) film with a unique geometry. All full‐wave simulations were carried out using the commercial software CST Microwave Studio with the frequency‐domain finite element method (FEM) solver, which is suitable for high‐precision simulation of periodic metamaterial structures and frequency‐dependent magnetic materials involved in this work. For the unit‐cell simulation of the proposed absorber, periodic boundary conditions (unit cell) were applied along the *x*‐ and *y*‐directions to model the infinite periodic extension of the metamaterial array. Two Floquet ports were set along the *z*‐direction (incident wave propagation direction), the upper port was configured as the excitation port to input transverse electric (TE) or transverse magnetic (TM) fundamental modes, and the lower port was set as the receiving port. The simulation frequency range was set to 0.1–4.0 GHz, which fully covers the target operating bandwidth of the absorber. In addition, an adaptive tetrahedral mesh scheme was adopted in the simulation, with the global mesh accuracy set to level 8, and local mesh encryption performed on the key functional regions including the resonant structure, magnetic material layer and dielectric substrate, to ensure the calculation accuracy of the electromagnetic field in the strong resonance region. The maximum mesh step was limited to 1/20 of the shortest operating wavelength, and we verified that when the mesh accuracy was higher than level 6, the deviation of the simulated absorption rate was less than 0.2%, which fully meets the simulation accuracy requirements. As for the convergence analysis of the results, we choose to rely on the built‐in convergence algorithm of the CST software. Specifically, we calculate the required absorptivity data using the *S*_11_ and *S*_21_ data obtained from simulation, the *S*_21_ will be zero due to the presence of the metal backplane. The absorption characteristics of each constituent ITO layer operating independently as a single‐layer absorber are shown in Figure 2b. Each exhibits a distinct, narrow single‐resonance response, with the peak absorption frequency ranging from 2.2 to 3.2 GHz depending on the specific ITO pattern and its conductivity. This variation provides essential design freedom for the integrated multi‐layer structure. The performance evolution from a single layer to the full three‐layer stack is summarized in Figure 2c,d. When only the bottom ITO layer is present, a weak resonance occurs at 3.2 GHz with less than 50% absorption. Integrating a second ITO layer shifts the primary resonance down to 1.2 GHz and introduces a second resonant mode, characteristic of a dual‐band response, while increasing the absorption rate beyond 90%. Finally, the complete three‐layer TLMA achieves a further redshift of the lowest resonance to approximately 0.6 GHz. As shown in Figure 2d, it exhibits a triple‐resonance characteristic, delivering over 90% absorption across a 300 MHz bandwidth from 0.62 to 0.92 GHz. This confirms the role of multi‐layer stacking in reducing the operational frequency. However, the absorption bandwidth remains narrow, highlighting a fundamental limitation of relying solely on structural resonance within a constrained electrical thickness.

**FIGURE 2:**
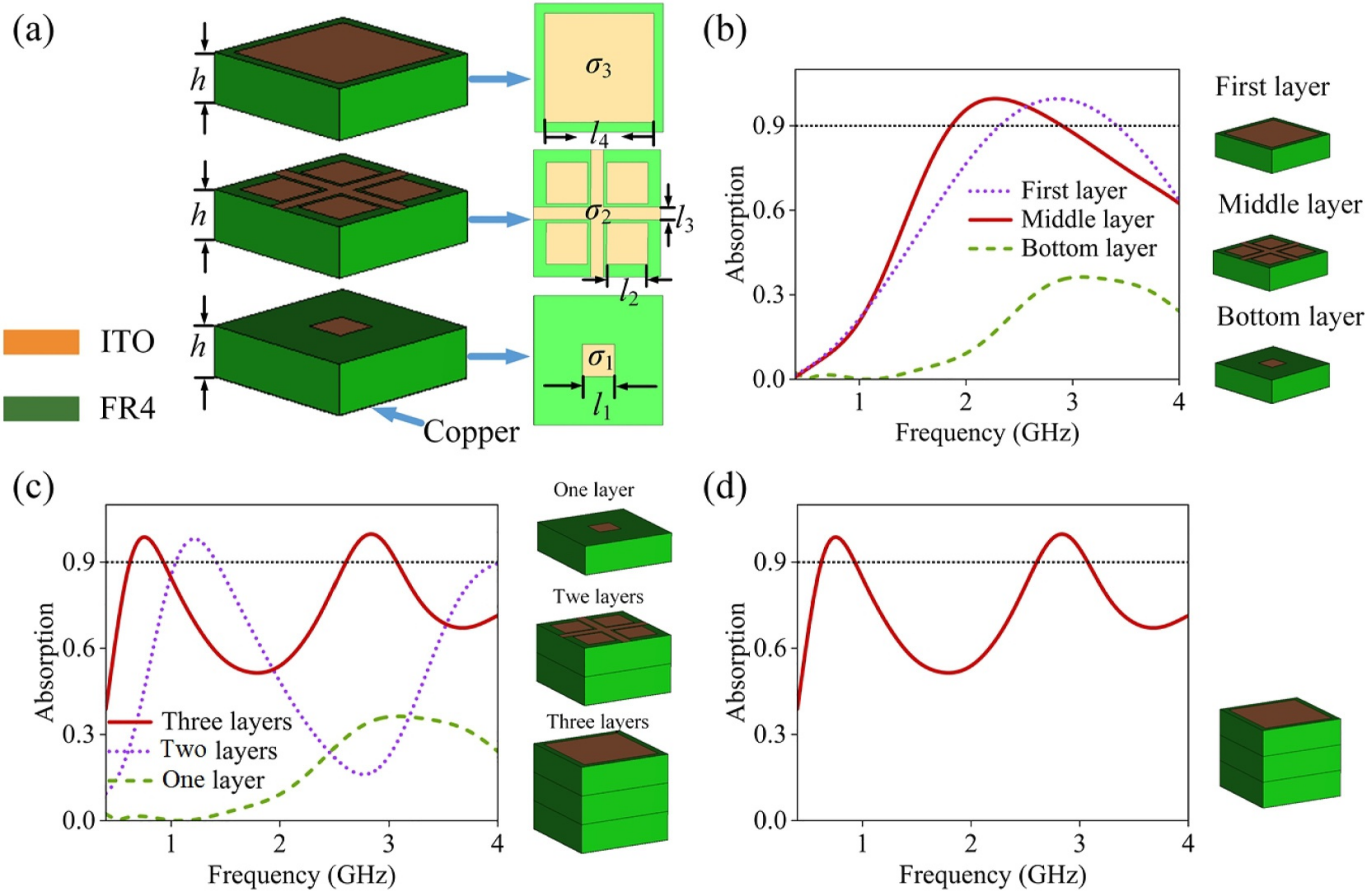
The schematic and design process of the TLMA. (a) Schematic diagram of the unit structure. (b) Absorption characteristics of the single‐layer resonant structures. (c) Absorption characteristics of multi‐layer resonant structures. (d) Simulation results of the TLMA.

To achieve optimal absorption performance, a systematic parameter study was conducted. The effects of key geometric and material parameters on the reflection coefficient of the TLMA are summarized in Supporting Information S1: Section A. Results in Supporting Information S1: Figure S1a,b indicate that increasing the side lengths of either the middle (*l*_2_) or top (*l*_4_) ITO patch results in a redshift of the fundamental resonance frequency. However, this low‐frequency shift is accompanied by a narrow absorption bandwidth. In contrast, variations in the electrical properties exhibit a different influence, as shown in Supporting Information S1: Figure S1c,d. Increasing the conductivity (*σ*_2_ and *σ*_3_) of these ITO patches primarily enhances the absorption efficiency within the low‐frequency band, with a less pronounced effect on the resonance frequency. This indicates that the geometric dimensions of the ITO patches are the primary drivers for tuning the operational frequency, whereas their conductivity is critical for controlling the absorption depth. The influence of other structural parameters was also investigated. Supporting Information S1: Figure S1e indicates that increasing the thickness (*h*) of each dielectric layer will induce a redshift of the resonance, yet this comes at the expense of an increased overall thickness. The unit cell period (*p*) exhibits a nonlinear effect; an optimal period exists for minimal operational frequency, with deviations to either larger or smaller values causing an upward frequency shift, as shown in Supporting Information S1: Figure S1f. Guided by these parametric relationships, the structure was optimized to balance low‐frequency operation, bandwidth, and thickness.

Despite the optimization described in the previous section, the TLMA alone, under a fixed thickness constraint, struggles to achieve a sufficiently wide operating bandwidth. To overcome this limitation, we propose to enhance its resonant characteristics by loading a MM layer, targeting improved absorption in the ultra‐low frequency regime. We first investigate the intrinsic absorption properties and parameter dependence of the MM itself. A standalone MM‐based metasurface unit cell was analyzed, as shown in Supporting Information S1: Figure S2a of Section B. It consists of a bottom copper ground plane and a MM layer with a period *p* = 14.0 mm. Supporting Information S1: Figure S2b shows that increasing the MM thickness *h*_1_ leads to a predictable redshift of its absorption resonance frequency. More critically, Supporting Information S1: Figure S2c,d illustrate the distinct roles of the complex permeability's real (*μ*′) and imaginary (*μ*″) parts. The real part *μ*′ predominantly governs the resonance frequency position, whereas the imaginary part *μ*″ directly controls the absorption efficiency across the band. This analysis provides clear design guidelines. For low‐frequency metasurface absorbers, selecting an MM with simultaneously high *μ*′ and *μ*″ values is crucial to concurrently lower the operational frequency and enhance the absorption efficiency. Furthermore, increasing the MM thickness within practical limits facilitates the excitation of lower‐frequency resonances.

To further elucidate the enhancement mechanism, we first investigate the integration of MM with single‐layer resonant structures. A conventional single‐layer resonant structure was designed as a baseline, with its unit cell depicted in Figure 3a. It consists of a square ITO patch (side length *l* = 30 mm, conductivity *σ*_1_ = 1.6 × 10^5^ S/m) on an FR4 dielectric substrate (period *p* = 40 mm, thickness *h* = 6 mm), backed by a copper ground plane. Its simulated absorption is plotted as the purple dotted line in Figure 3c. This structure exhibits a typical narrowband, single‐resonance response, with a peak absorption of 94% at 5.3 GHz and a −10 dB bandwidth (absorption > 90%) of only 400 MHz (5.1–5.5 GHz). Subsequently, a 1‐mm‐thick (*h*_1_) layer of the MM named “ECCOSORB SF 11.0–18.0” was added on top of this single‐layer structure, forming the composite unit cell shown in Figure 3b. The absorption response of this modified structure, shown by the red solid line in Figure 3c, demonstrates a dramatic improvement. The resonance is significantly enhanced, with the peak absorption increasing to 99.99%, and concurrently experiences a distinct low‐frequency shift to 4.5 GHz. The above analysis validates that the loading of MM can profoundly intensify the resonance strength and effectively promote a lower operational frequency in resonant absorbers. This key finding directly informs and motivates our subsequent design of a broadband, low‐frequency absorber by integrating MM with the TLMA.

**FIGURE 3:**
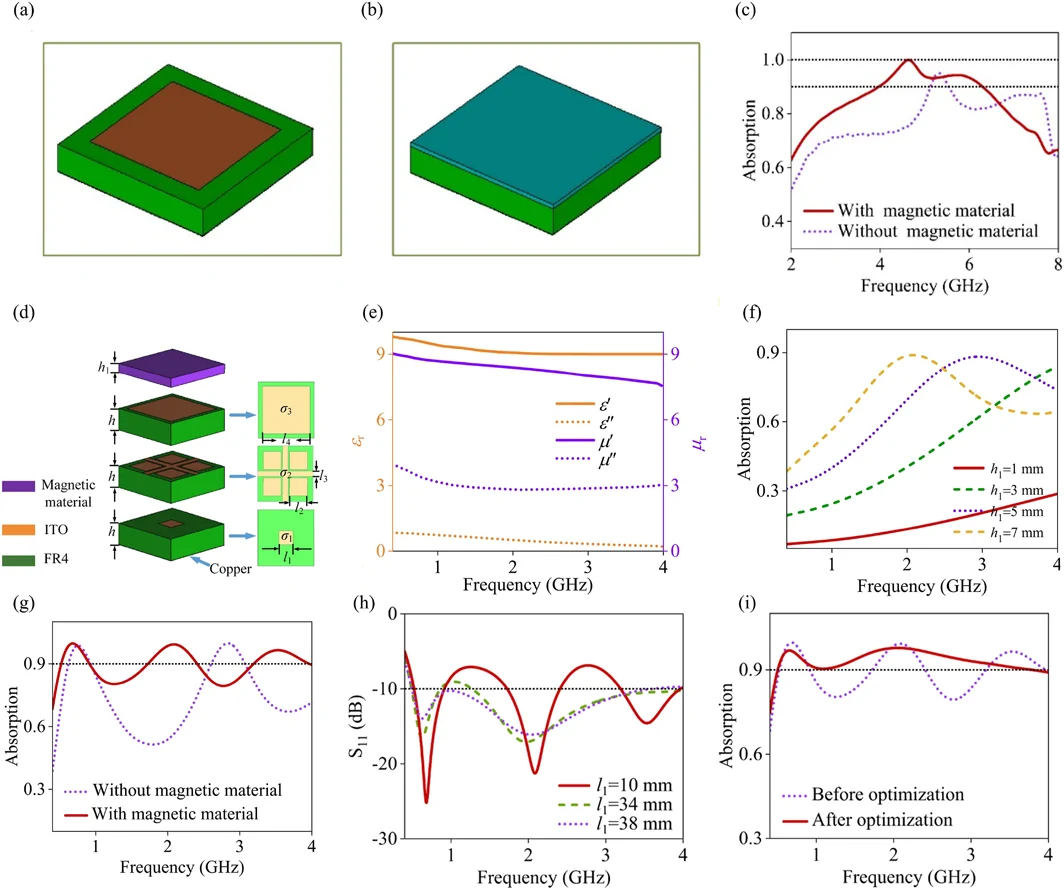
Performance of the composite meta‐absorber. Structural diagram of (a) the single‐layer resonant structure and (b) the unit cell after loading MM. (c) Comparison of the enhancement effect before and after loading MM. (d) Structural diagram of the unit cell. (e) Trend of complex electromagnetic parameters of MM. Comparison of absorption performance of (f) MM with different thicknesses and (g) before and after loading MM. (h) Influence of the size *l*_1_. (i) Comparison of absorption rates before and after optimization. The final parameters are set as *σ*_1_ = 1.6 × 10^5^ S/m, *σ*_2_ = 2.0 × 10^5^ S/m, *σ*_3_ = 6.0 × 10^5^ S/m, *l*_1_ = 38.0 mm, *l*_2_ = 13.0 mm, *l*_3_ = 4.0 mm, *l*_4_ = 32.0 mm, *h* = 12.0 mm, *h*_1_ = 5.0 mm.

Building on the analysis of the TLMA and MM loaded in the previous sections, we now present the design of the final composite absorber. As illustrated in Figure 3d, the MM named “ECCOSORB SF 11.0–18.0” is integrated as a top layer onto the previously optimized TLMA. The choice of this MM is deliberate: its complex electromagnetic parameters, plotted in Figure 3e, reveal simultaneously high real and imaginary parts across a broad frequency range. The high real part is conducive to lowering the operational frequency, whereas the high imaginary part is paramount for ensuring strong magnetic loss and high absorption efficiency. The thickness of the MM layer (*h*_1_) is a critical parameter. Only with a MM layer, Figure 3f shows that increasing *h*_1_ enhances absorption and redshifts the resonance, albeit at the cost of greater overall thickness. A thickness of 5 mm was selected as an optimal trade‐off between performance and the ultra‐thin profile objective. The initial absorption performance of the combined structure is shown in Figure 3g. Although a significant broadband improvement over the TLMA is evident, the absorption in certain bands remains below 90%. This indicates that simply stacking the MM onto the TLMA is insufficient for perfect impedance matching across the entire target band. Therefore, a final stage of parameter optimization is essential to fine‐tune the coupling between the MM layer and the TLMA, aiming to achieve uniformly high absorption in a broad band. To address the sub‐optimal absorption noted in the initial design, a final parameter optimization was performed. This process identified the dimension of the bottom ITO patch (*l*_1_) as a particularly sensitive parameter for achieving broadband impedance matching. As shown in Figure 3h, varying *l*_1_ yields distinct absorption profiles. A small *l*_1_ results in overly strong, localized resonances, impairing broadband impedance matching. At *l*_1_ = 10 mm, three distinct resonance peaks emerge, with high absorption at each peak but degraded performance in the intervening frequencies. As *l*_1_ is progressively increased, the resonance strength moderates, leading to a more balanced and continuous absorption response across the band. An optimal condition is reached at *l*_1_ = 38 mm, where the reflectivity remains below −10 dB (corresponding to > 90% absorption) across the entire 0.5–3.8 GHz range. The efficacy of this optimization is clearly demonstrated in Figure 3i. Compared to the pre‐optimized response, the final design exhibits significantly enhanced and consistently high absorption efficiency, surpassing 90% throughout the target ultra‐wide band.

The optimized composite absorber demonstrates excellent performance, which can be understood through an analysis of its surface current distributions. Figure 4a locates the five representative frequencies (0.5, 0.78, 1.5, 2.5, and 3.5 GHz) on the absorption curve, whereas Figure 4b–f compare the surface current densities of the standalone TLMA (left) and the final composite absorber (right) at these points. The comparison reveals a consistent and powerful enhancement mechanism. At the lowest frequency (0.5 GHz, Figure 4b), the loaded MM participates directly in the resonance, forming a strong magnetic resonance loop with the top ITO layer and the ground plane. This is evidenced by a significant increase in surface current density compared to the TLMA alone. The same pronounced enhancement is observed at 0.78 GHz (Figure 4c). At 1.5 GHz (Figure 4d), where the second and third ITO layers collectively act as a ground plane for the top layer, the MM loading again intensifies the resonant currents. This trend continues at higher frequencies. At 2.5 GHz (Figure 4e), resonance occurs between the top/second layers and the third layer, and at 3.5 GHz (Figure 4f), between the top and second layers. In all cases, the integration of the MM markedly amplifies the surface current. This comprehensive analysis leads to a clear physical interpretation: the magnetic material layer does not merely add loss; it actively couples to and enhances the intrinsic resonances of the TLMA across the entire bandwidth. By intensifying these resonant currents, the structure dissipates significantly more electromagnetic energy as ohmic loss, which directly translates to the observed increase in absorption rate and the expansion of the absorption bandwidth to over 90% from 0.5 to 3.8 GHz.

**FIGURE 4:**
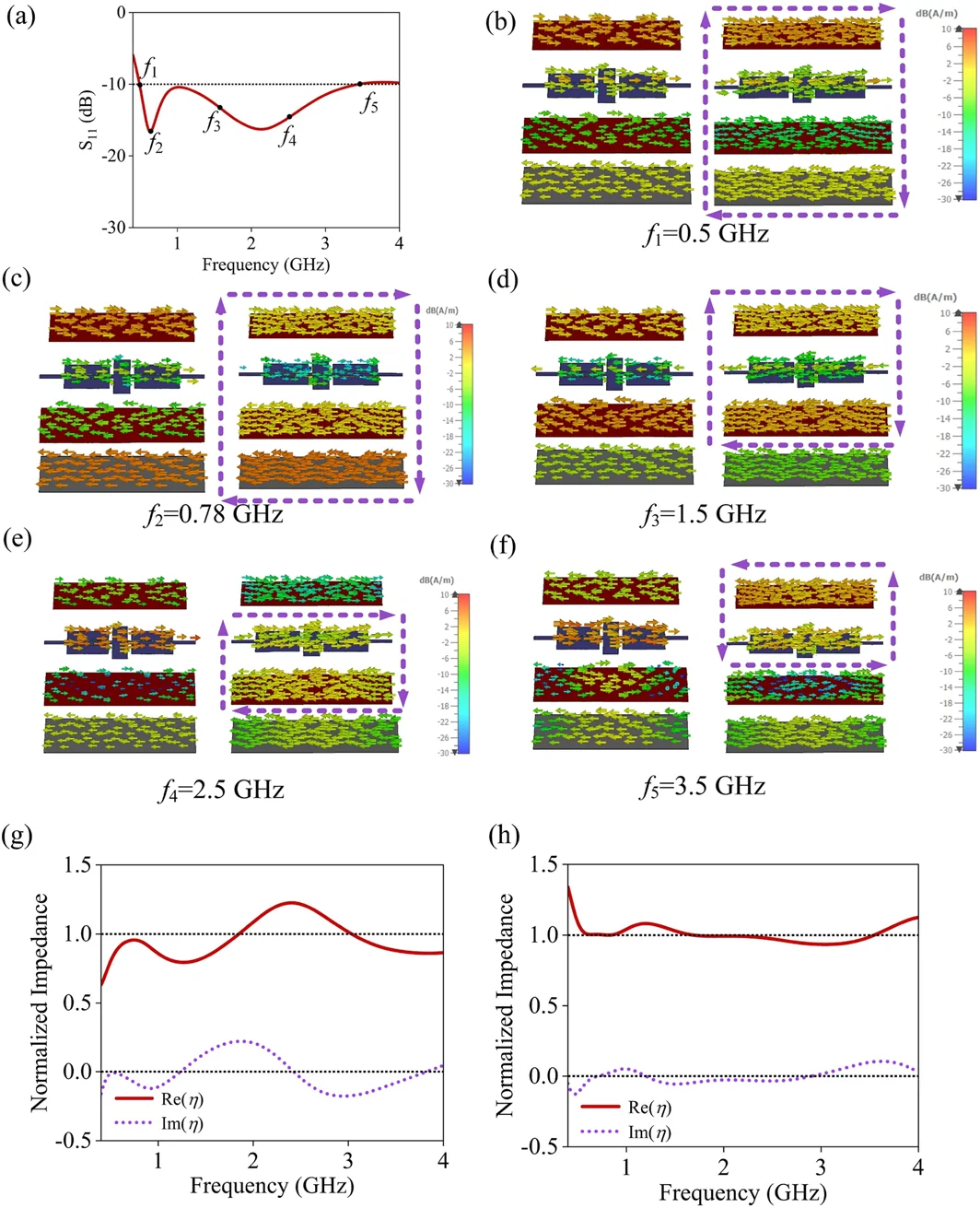
The comparison of the current distributions and normalized equivalent impedance for the structure with or without the MM layer. (a) The five representative frequencies on the absorption curve. Surface current distributions of the TLMA (left) and the composite absorber (right) at frequencies of (b) 0.5 GHz, (c) 0.78 GHz, (d) 1.5 GHz, (e) 2.5 GHz, and (f) 3.5 GHz. Curves of the normalized equivalent impedance of the TLMA (g) without loading MM and (h) with loading MM.

To explicitly demonstrate the regulatory role of the MM, we calculated the normalized impedance [43] *Z*_eff_/*Z*_0_ for both the standalone TLMA and the final MM‐loaded composite meta‐absorber. The normalized impedance is the ratio of the input equivalent impedance of the absorber to the free space wave impedance, which is the physical parameter determining the absorptivity of the absorber. The results are plotted in Figure 4g,h, respectively. Figure 4g reveals that the impedance of the TLMA alone fluctuates significantly across the band. Both its real and imaginary parts deviate considerably from the ideal values of 1 and 0, explaining its inherent narrowband limitation. In stark contrast, the impedance of the composite absorber (Figure 4h) is remarkably stabilized. Its real part remains close to 1, and the imaginary part near 0 across the entire 0.5–3.8 GHz band, indicating near‐perfect matching to free space. This comparative analysis confirms the pivotal function of the MM, which actively tunes and stabilizes the overall effective impedance of the system. By compensating for the TLMA's inherent impedance mismatch, the MM enables the broadband impedance matching that is essential for achieving the observed ultra‐wideband, high‐efficiency absorption.

To validate the performance of the proposed absorber, a prototype was fabricated and characterized. The sample, with overall dimensions of 400 × 400 × 36 mm, comprises a 10 × 10 array of the unit cells. As shown in Figure 5a, it was assembled by fixing a copper ground plane to the bottom of the fabricated TLMA and adhering the 5‐mm‐thick MM layer on top. The far‐field scattering and absorption performance were measured in an anechoic chamber. Due to equipment limitations, the full band (0.5–3.8 GHz) was assessed in two sub‐bands using different antenna setups. For the 1.0–3.8 GHz band, two standard‐gain broadband double‐ridged horn antennas were connected to a vector network analyzer (AV3672B) as transmitter and receiver. The sample was mounted on the turntable, as shown in the setup of Figure 5b. Measurements were taken at 57 frequency points with a 0.05 GHz interval. At each point, the angular scattering profiles of both the sample and an equal‐sized copper reference plate were recorded. The absorption at each frequency was then calculated from the ratio of their total integrated scattered power [44, 45, 46, 47]. The results are plotted in Figure 5c. For the lower 0.5–1.0 GHz band, a custom‐designed array feed source was employed for excitation and reception, as shown in the Figure 5d,e, following the same VNA‐based measurement and calculation procedure at 11 discrete points. The resulting absorption is shown in Figure 5f. The experimental results show good agreement with the simulation. The absorption exceeds 90% across most of the 0.5–3.8 GHz band. Minor discrepancies are observed, notably at individual points in the 0.5–1.0 GHz band (with a minimum of 83%) and within the 1.0–1.3 GHz sub‐band (absorption > 86%). These deviations are primarily attributed to imperfect bonding between the MM and TLMA layers and slight variations in the fabricated ITO conductivity compared to the simulation model. To conclusively verify that the reduction in reflected power is due to absorption rather than diffuse scattering, the far‐field radiation patterns were compared. Figure 5g–i present the normalized scattering patterns of the sample and the metal reference at three representative frequencies (0.5, 2.0, and 3.5 GHz). A significant reduction (> 10 dB) in the specular reflection lobe (the main radiation direction) is observed for the absorber at all frequencies, confirming that incident energy is effectively dissipated within the structure.

**FIGURE 5:**
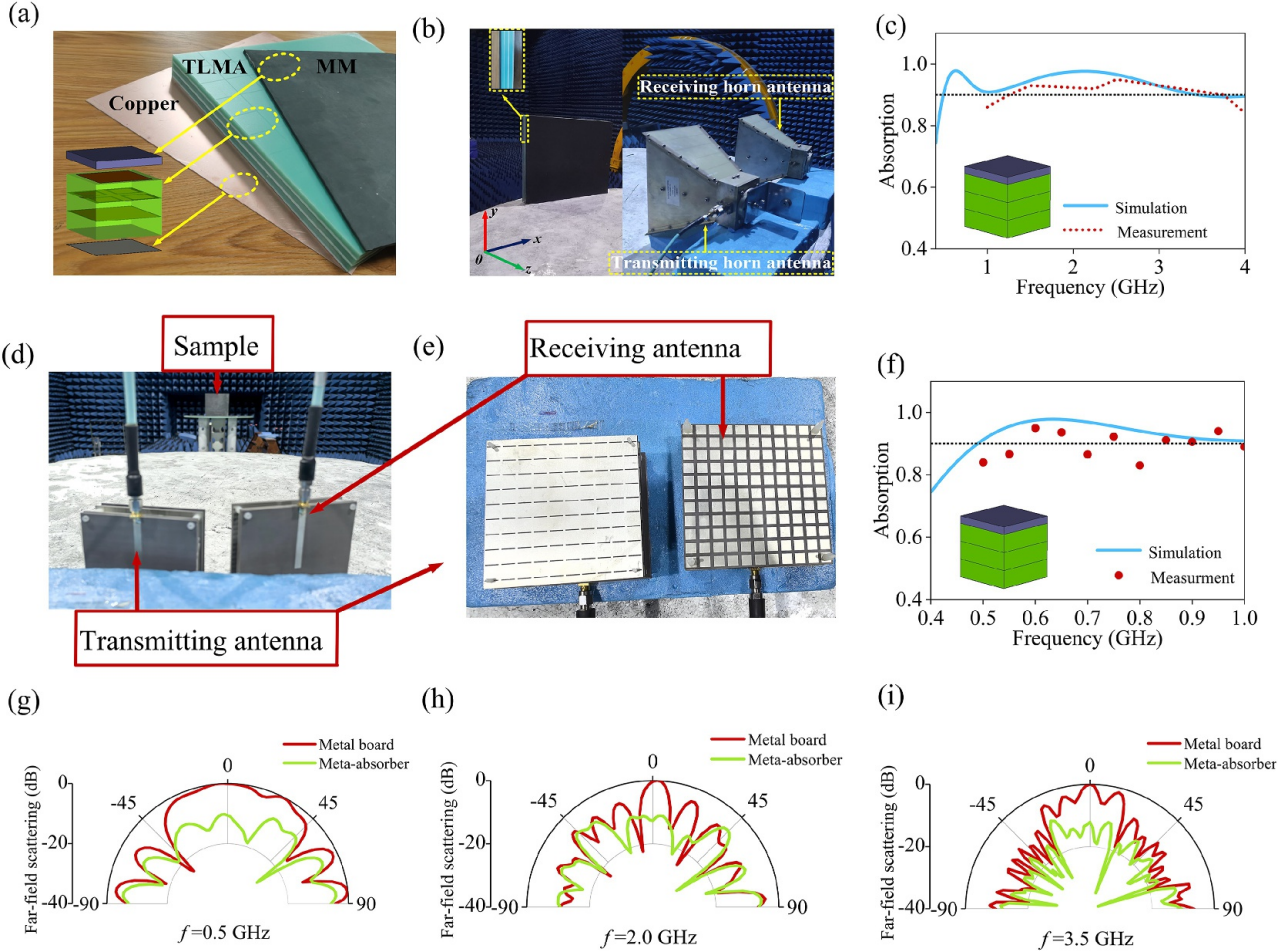
Experimental results of the composite meta‐absorber. (a) Sample structure diagram. (b) Construction of the far‐field test environment. (c) Comparison of experimental and simulated absorption curves for 1.0–3.8 GHz. (d–e) Construction of the far‐field test environment for 0.5–1.0 GHz. (f) Comparison of experimental and simulated absorption curves for 0.5–1.0 GHz. Comparison of scattering between metal plates and composite metamaterial absorber at (g) 0.5 GHz, (h) 2.0 GHz, and (i) 3.5 GHz.

The performance of the absorber under different polarization states and oblique incidence angles was further investigated, as summarized in Supporting Information S1: Figure S3a of Section C. Supporting Information S1: Figures S3a,b present the absorption spectra for transverse electric (TE) and transverse magnetic (TM) polarized waves at various incident angles (*θ*). A clear distinction is observed that for TE polarization, the absorber can maintain a stable absorptivity above 90% when the incident angle *θ* is up to 30°, whereas for TM polarization, this stable angular range extends to *θ* = 60°. The oblique incidence absorption performance of the absorber is fundamentally determined by the impedance matching between free space and the absorber surface. There are essential differences between TE and TM polarizations in the variation law of wave impedance under oblique incidence and the coupling characteristics with the metasurface structure, which finally lead to the difference in their angular dependence. For oblique incidence of TE polarization, the electric field is parallel to the metasurface plane, and the equivalent wave impedance of free space rises in the law of *ƞ*_0_/cos*θ* with the increase of incident angle *θ*. The free space wave impedance rises with the increase of incident angle, whereas the equivalent impedance of the structure almost remains unchanged, leading to a sharp aggravation of impedance mismatch and rapid degradation of absorption performance, thus it is highly sensitive to incident angle. For TM polarization under oblique incidence, the magnetic field is parallel to the metasurface plane, the normal component of the electric field can maintain stable coupling with the structure, and the equivalent wave impedance of free space decreases in the law of *ƞ*_0_·cos*θ* with the increase of incident angle *θ*. The equivalent impedance decreases synchronously with the increase of incident angle, which matches the variation trend of free space wave impedance. Therefore, the impedance matching degrades slowly and the angular robustness is better.

To quantify this trend, the average absorption across the entire operating band (0.5–3.8 GHz) was calculated for each angle and polarization, as plotted in Supporting Information S1: Figure S3c. The TM polarization maintains an average absorption above 90% for angles up to 60°, confirming its robust angular stability. In contrast, the average absorption for TE polarization declines to approximately 82% at *θ* = 45°. Furthermore, the structure's response to in‐plane polarization rotation was evaluated. Supporting Information S1: Figure S3d shows that the absorption remains virtually unchanged for all polarization angles (*φ*). This excellent polarization insensitivity is a direct consequence of the four‐fold rotational symmetry of the unit cell design. In summary, the proposed absorber exhibits good stability under oblique incidence, particularly for TM polarization, and is essentially insensitive to the polarization state of normally incident waves. These characteristics affirm its practical utility for real‐world applications where wave incidence may be non‐ideal.

In addition, we added Table 1: Performance comparison between this work and recently reported metamaterial absorbers, which covers indicators including operating frequency range, fractional bandwidth, normalized thickness, absorption efficiency, etc. The advantages and performance trade‐offs of the proposed design are analyzed based on the comparison results. The comparison results show that this work achieves a significant breakthrough in the comprehensive performance of low‐frequency ultra‐wideband absorption and ultra‐thin thickness. It simultaneously realizes an ultra‐wide fractional bandwidth of 153.5% and an ultra‐thin normalized thickness of 0.068λ, fully covering the P, L and S bands, which solves the pain point that the bandwidth and thickness of traditional low‐frequency absorbers are difficult to balance.

**TABLE 1:** Performance comparison between this work and recently reported metamaterial absorbers.

Reference	Operating frequency range (GHz)	Fractional bandwidth (%)	Normalized thickness (*λ*@ lowest frequency)	Absorption efficiency
[48]	5.8–27.3	129.9	0.12	> 90%
[49]	4–18	127.3	0.29	> 89%
[50]	4.72–8.49	80	0.07	> 90%
[51]	27–31.6	6.8	0.05	> 90%
This work	0.5–3.8	153.5	0.068	> 90%

## 3 Conclusion

In summary, we have proposed, fabricated, and experimentally validated an ultra‐thin composite meta‐absorber for high‐performance, ultra‐wideband operation in the low‐frequency regime. The design synergistically integrates a tailored TLMA with a top‐loaded MM layer. The TLMA establishes multiple resonances as a foundation, whereas the MM layer critically enhances and broadens these resonances through magnetic loss and optimized impedance matching. This magnetic‐material‐assisted resonance engineering enables the final structure, with a remarkably small electrical thickness of 0.068*λ*, to achieve over 90% absorption across an ultra‐wide bandwidth of 0.5–3.8 GHz (fractional bandwidth of 153.5%), effectively covering P to S bands. Both simulation and measurement results confirm the design's effectiveness, polarization insensitivity, and reasonable angular stability. The comparative analysis demonstrates its advantages in simultaneously achieving lower frequency, wider bandwidth, and thinner profile compared to existing designs. This work overcomes the typical low‐frequency trade‐off between thickness and bandwidth, providing a practical and effective strategy for advanced stealth and electromagnetic compatibility applications.

## Author Contributions

D.L. performed the conceptualization, modeling, and wrote the manuscript draft. X.C. and Y.C. conducted the S‐parameter measurements, far‐field measurements and explored measurement approaches. X.L. and X.L. were responsible for the investigation, review, and editing. Z.W performed the methodology and editing. F.G. reviewed and edited the manuscript. T.C. supervised the work, wrote, reviewed, and edited the manuscript.

## Funding

This work was supported by the National Natural Science Foundation of China (62371467, 62071423), Shaanxi Provincial Science Fund for Distinguished Young Scholars (2025JC‐JCQN‐086), the Key Research and Development Program of Zhejiang Province (2024C01160) and the Natural Science Foundation of Zhejiang Province (LR23F010004).

## Consent

Informed consent was obtained from all individuals included in this study.

## Conflicts of Interest

The authors declare no conflicts of interest.

## Supporting information


Supporting Information S1

## Data Availability

The data that support the findings of this study are available from the corresponding author upon reasonable request.
