# Supplementary material for: Low‐Frequency, Ultra‐Thin and Ultra‐Wideband Meta‐Absorber via Resonance Enhancement Harnessing Magnetic Material
Source: Nanophotonics. 2026 May 19;15(10):e70140. doi: 10.1002/nap2.70140 (PMC13642117; doi:10.1002/nap2.70140)
Supplement: Supplementary file 1 [file NAP2-15-e70140-s001.docx]

**Supporting Materials**

Low-Frequency, Ultra-thin and Ultra-Wideband Meta-Absorber via Resonance Enhancement Harnessing Magnetic Material

*Dan Liu*^1, 4^*, Xingshuo Cui*^2, 4^*, Yuxu Chen*^2, 4^*, Xiongyu Liang*^2^*, Xiaochun Liu*^3^*, Zhenxu Wang*^2,^ *^*^, Fei Gao*^1,^ *^*^, and Tong Cai*^1, 2,^ *^*^*

*^1^State Key Laboratory of Modern Optical Instrumentation, the Electromagnetics Academy Zhejiang University, 310027, Hangzhou, China*

*^2^Air and Missile Defense College, Air Force Engineering University, 710051, Xi’an, China*

*^3^The Research Institute for Special Structures of Aeronautical Composite ACICA, The Aeronautical Science Key Lab for High Performance Electromagnetic Windows, 250023, Ji’nan, China*

*^4^These authors contributed equally*

*Corresponding Authors: wzx206@126.com (Zhenxu Wang), caitong326@sina.cn (Tong Cai), gaofeizju@zju.edu.cn (Fei Gao)*

**Section A Parameter optimization process of the TLMA.**

**Figure S1** Parameter optimization process of the TLMA. (a-b) The influence of side lengths *l*_2_ and *l*_4_; (c-d) The influence of conductivities *σ*_2_ and *σ*_3_; (e) The influence of thickness *h;* (f) The influence of period *p*. The final parameters of TLMA are determined as: *σ*_1_=1.6×10^5^ S/m, *σ*_2_=2.0×10^5^ S/m, *σ*_3_=6.0×10^5^ S/m, *l*_1_=10.0 mm, *l*_2_=13.0 mm, *l*_3_=4.0 mm, *l*_4_=32.0 mm, *h*=12.0 mm.

**Section B The absorptive performance of the magnetic material.**

**Figure S2** Influence of MM parameters on absorption performance. (a) Unit cell of MM-based metasurface; (b) Influence of the thickness of MM-based metasurface. (c-d) Influence of the real and imaginary parts of the magnetic permeability of MM-based metasurface. The parameters of MM layer are set as *p* = 14.0 mm, *ε_r_=*9+0.01*i, and μ_r_=μ*_1_+*i μ*_2_*.*

**Section C The performance of the absorber under different polarization states and oblique incidence angles.**

**Figure S3** Performance of composite meta-absorber under different incident angles and different polarizations. Absorption performance at different incident angles under (a) TE and (b) TM modes. (c) Variation trend of average absorption efficiency with incident angle within the operating frequency band under TE and TM modes. (d) Comparison of absorption performance at different polarization angles.
